# Geodesic motion in Euclidean Schwarzschild geometry

**DOI:** 10.1140/epjc/s10052-022-11070-w

**Published:** 2022-12-02

**Authors:** Emmanuele Battista, Giampiero Esposito

**Affiliations:** 1 Department of Physics, University of Vienna, Boltzmanngasse 5, 1090 Vienna, Austria; 2 Dipartimento di Fisica “Ettore Pancini”, Complesso Universitario di Monte S. Angelo, Università degli Studi di Napoli “Federico II”, Via Cintia Edificio 6, 80126 Naples, Italy; 3 Istituto Nazionale di Fisica Nucleare, Sezione di Napoli, Complesso Universitario di Monte S. Angelo, Via Cintia Edificio 6, 80126 Naples, Italy

## Abstract

This paper performs a systematic investigation of geodesic motion in Euclidean Schwarzschild geometry, which is studied in the equatorial plane. The explicit form of geodesic motion is obtained in terms of incomplete elliptic integrals of first, second and third kind. No elliptic-like orbits exist in Euclidean Schwarzschild geometry, unlike the corresponding Lorentzian pattern. Among unbounded orbits, only unbounded first-kind orbits are allowed, unlike general relativity where unbounded second-kind orbits are always allowed.

## Introduction

Ever since Schwarzschild obtained his spherically symmetric solution of vacuum Einstein equations [1], the resulting spacetime geometry has been investigated with a huge variety of perspectives. In particular, we find it important to mention the following works.

(i) The Regge–Wheeler proof [2] that a Schwarzschild singularity will undergo small vibrations about the spherical form and will therefore remain stable if subjected to a small nonspherical perturbation.
(ii) The detailed investigation of geodesic motion in the case of Lorentzian signature of the metric performed in Refs. [3–6], as well as the more recent works regarding Schwarzschild–(anti-)de Sitter spacetimes, BTZ black holes, noncommutative Schwarzschild black holes, and static and spherically symmetric traversable wormholes geometries [7–10].
(iii) The proof in Ref. [11] that general vacuum initial data with no symmetry assumed, if sufficiently close to Schwarzschild data, evolve to a vacuum spacetime which possesses a complete future null infinity, remains close to Schwarzschild in its exterior, and approaches a member of the Schwarzschild family as an appropriate notion of time goes to infinity.
(iv) The work on gravitational instantons in Euclidean quantum gravity [12], until the recent discovery of a new asymptotically flat instanton [13], and the even more recent proof that all known gravitational instantons are Hermitian [14].
(v) A broader set of investigations in Euclidean Schwarzschild, including zero modes [15], black holes in matrix theory [16], Yang–Mills solutions [17, 18], the master equations of a static perturbation [19], multiplicative noise [20].
(vi) The work by the authors in Ref. [21], where a basic integral formula of geometric measure theory has been evaluated explicitly in the relevant case of Euclidean Schwarzschild geometry, and it has been suggested that the in-out amplitude for Euclidean quantum gravity should be evaluated over finite-perimeter Riemannian geometries that match the assigned data on their reduced boundary. This work has also obtained a heuristic derivation of a formula expressing a correction to the classical entropy of a Schwarzschild black hole. Furthermore, in Ref. [22] we have provided explicit examples for the concept of generalized discontinuous normals to finite-perimeter sets in non-Euclidean spaces and two-dimensional gravity settings.

Motivated by our original calculations in Refs. [21, 22], in this paper we study geodesic motion in Euclidean Schwarzschild geometry. The present work can be seen as a step towards a novel perspective on some features of classical and quantum Euclidean gravity. From the point of view of functional-integral quantization, the work in Refs. [21, 22] has in our opinion good potentialities because measurable sets belong to two broad families: either they have finite perimeter, or they do not. In the former case, the tools of geometric measure theory [23] might help in putting on firm ground the so far purely formal work of theoretical physics literature.

If one tries to understand the very nature of quantum field theory, one may still regard it as integration over suitable function spaces [24], at least in order to define and evaluate in-out amplitudes. This involves the action functional and the effective action, and is therefore a part of the relativistically invariant, space-time approach to quantum field theory [25, 26]. The Euclidean approach is a mathematical framework where this form of quantization acquires a mathematical meaning and is therefore physically relevant, despite the fact that the space-time metric has Lorentzian (rather than Riemannian) signature. For example, one first solves a heat equation for a suitable Green function, and its analytic continuation yields eventually the Feynman propagator.

Gravitational instantons play a role in the tree-level evaluation of quantum amplitudes, and their investigation in the seventies led also to new results in Riemannian geometry [27].

In recent years, some authors have considered a novel geometric perspective on the nature of particles. When compact gravitational instantons are studied, it turns out that the neutron can be described by complex projective space $CP^{2}$ [28] with the associated Fubini-Study metric, but more recently [29, 30], asymptotically flat instantons such as Euclidean Schwarzschild have been considered as candidates for a geometric description of the neutron. Although none of these arguments is compelling, they add evidence in favour of gravitational instantons having good potentialities, if one is interested in foundational and qualitative features of the laws of nature.

Moreover, the systematic proof of geodesic completeness of gravitational instantons as a possible criterion for their singularity-free nature has not been attempted nor obtained in the literature, as far as we know. This would be of interest both in mathematical and in theoretical physics of fundamental interactions.

The paper is organized as follows. Section 2 obtains the equations for geodesic motion in the equatorial plane. Section 3 solves the cubic equation for turning points and provides a qualitative analysis of the orbits, whereas the explicit solution in terms of elliptic integrals jointly with its graphical representation is obtained in Sect. 4. The lack of circular orbits is proved in Sect. 5. Concluding remarks are made in Sect. 6, and relevant details are given in the appendices.

## Geodesic equations in Euclidean Schwarzschild geometry

The Euclidean Schwarzschild metric expressed in Schwarzschild coordinates $(\tau ,r,\theta ,\phi )$ reads as [31–33]

2.1 $$\begin{aligned} g_E^{(1)}= & {} \left( 1-\frac{2M}{r}\right) {\textrm{d}} \tau \otimes {\textrm{d}} \tau + \dfrac{{\textrm{d}} r \otimes {\textrm{d}} r}{\left( 1-\frac{2M}{r}\right) } \nonumber \\{} & {} + r^2 \left( {\textrm{d}} \theta \otimes {\textrm{d}} \theta + \sin ^2 \theta \; {\textrm{d}} \phi \otimes {\textrm{d}} \phi \right) , \end{aligned}$$

where the link with the Lorentzian-signature metric is obtained by setting $\tau = \textrm{i}t$. We work on the real Riemannian section where the metric is positive-definite. This implies that the *r* coordinate must obey the restriction

2.2 $$\begin{aligned} r \ge 2M, \end{aligned}$$

which agrees with the restriction obtained on using Kruskal–Szekeres coordinates. Thus, the Kretschmann invariant $R^{\mu \nu \sigma \rho } R_{\mu \nu \sigma \rho }$ is a bounded function on the real Riemannian section of Euclidean Schwarzschild.

By exploiting the symmetries of Schwarzschild geometry, we can limit our investigation to the equatorial plane $\theta = \pi /2$, where the geodesic equations read as

2.3a $$\begin{aligned}&\dfrac{{\textrm{d}}^2 r }{{\textrm{d}} \uplambda ^2 } - \dfrac{A^\prime }{2A} \left( \dfrac{{\textrm{d}} r }{{\textrm{d}} \uplambda }\right) ^2 -rA \left( \dfrac{{\textrm{d}} \phi }{{\textrm{d}} \uplambda }\right) ^2 -{AA' \over 2} \left( \dfrac{{\textrm{d}} \tau }{{\textrm{d}} \uplambda }\right) ^2=0, \end{aligned}$$

2.3b $$\begin{aligned}&\dfrac{{\textrm{d}}^2 \phi }{{\textrm{d}} \uplambda ^2 } +\dfrac{2}{r} \dfrac{{\textrm{d}} r }{{\textrm{d}} \uplambda } \dfrac{{\textrm{d}} \phi }{{\textrm{d}} \uplambda } =0, \end{aligned}$$

2.3c $$\begin{aligned}&\dfrac{{\textrm{d}}^2 \tau }{{\textrm{d}} \uplambda ^2 } +\dfrac{A^\prime }{A} \dfrac{{\textrm{d}} r }{{\textrm{d}} \uplambda } \dfrac{{\textrm{d}} \tau }{{\textrm{d}} \uplambda } =0, \end{aligned}$$

where $\uplambda$ is the affine parameter, the prime denotes the derivative with respect to the *r* variable and we have set

2.4 $$\begin{aligned} A(r) \equiv 1-{2M \over r}. \end{aligned}$$

After dividing Eqs. (2.3b) and (2.3c) by ${\textrm{d}}\phi /{\textrm{d}}\uplambda$ and ${\textrm{d}}\tau /{\textrm{d}}\uplambda$, respectively, we obtain

2.5 $$\begin{aligned} \dfrac{{\textrm{d}}}{{\textrm{d}}\uplambda } \left[ \log \left( \dfrac{{\textrm{d}}\phi }{{\textrm{d}}\uplambda } \right) + \log r^2 \right]&=0, \end{aligned}$$

2.6 $$\begin{aligned} \dfrac{{\textrm{d}}}{{\textrm{d}}\uplambda } \left[ \log \left( \dfrac{{\textrm{d}}\tau }{{\textrm{d}}\uplambda } \right) + \log A \right]&=0, \end{aligned}$$

from which we derive

2.7 $$\begin{aligned} \dfrac{{\textrm{d}} \tau }{{\textrm{d}}\uplambda }&=\dfrac{C}{A(r(\uplambda ))} \end{aligned}$$

2.8 $$\begin{aligned} r^2 \dfrac{{\textrm{d}} \phi }{{\textrm{d}}\uplambda }&=J, \end{aligned}$$

*C* and *J* being integration constants. By virtue of Eqs. (2.7) and (2.8), Eq. (2.3a) reads as

2.9 $$\begin{aligned} \dfrac{{\textrm{d}} }{{\textrm{d}}\uplambda } \left[ A^{-1}(r(\uplambda )) \left( \left( \dfrac{{\textrm{d}} r}{{\textrm{d}}\uplambda }\right) ^2 +C^{2}\right) +\dfrac{J^2}{r^2}\right] =0, \end{aligned}$$

and hence we arrive at

2.10 $$\begin{aligned} A^{-1}(r(\uplambda )) \left[ \left( \dfrac{{\textrm{d}} r}{{\textrm{d}}\uplambda }\right) ^2 +C^{2}\right] +\dfrac{J^2}{r^2}= {\mathcal {E}}, \end{aligned}$$

where ${\mathcal {E}}>0$ is a constant.

The squared line element evaluated via (2.1) and with $\theta =\pi /2$ reads as

2.11 $$\begin{aligned} \left. {\textrm{d}}s^2 \right| _{\theta =\pi /2} = A(r) {\textrm{d}}\tau ^2 + A^{-1}(r) {\textrm{d}}r^2 + r^2 {\textrm{d}}\phi ^2, \end{aligned}$$

then from Eqs. (2.7), (2.8), and (2.10) we obtain the useful relation

2.12 $$\begin{aligned} {\textrm{d}}s^2 = {\mathcal {E}} {\textrm{d}}\uplambda ^2, \end{aligned}$$

which makes it possible to write the equations defining geodesic motion as

2.13a $$\begin{aligned} \left( \dfrac{{\textrm{d}}r }{{\textrm{d}}s} \right) ^2&=\left( 1-\dfrac{2M}{r}\right) \left( 1-\dfrac{L^2}{r^2}\right) - C^{2} E^2, \end{aligned}$$

2.13b $$\begin{aligned} \dfrac{{\textrm{d}}\phi }{{\textrm{d}}s}&=\dfrac{L}{r^2}, \end{aligned}$$

2.13c $$\begin{aligned} \dfrac{{\textrm{d}}\tau }{{\textrm{d}}s}&=\dfrac{CE}{\left( 1-\dfrac{2M}{r}\right) }, \end{aligned}$$

where we have defined the real-valued constants *E* and *L* as

2.14a $$\begin{aligned} E&\equiv \dfrac{1}{\sqrt{{\mathcal {E}}}}, \end{aligned}$$

2.14b $$\begin{aligned} L&\equiv \dfrac{J}{\sqrt{{\mathcal {E}}}}=JE. \end{aligned}$$

Upon introducing the variable

2.15 $$\begin{aligned} u={1 \over r}, \end{aligned}$$

Eq. (2.13) can be equivalently written as

2.16 $$\begin{aligned} \left( {{\textrm{d}}u \over {\textrm{d}}\phi }\right) ^2&= {1 \over L^{2}}\left( {dr \over ds}\right) ^{2}= {\mathcal {F}}(u), \end{aligned}$$

2.17 $$\begin{aligned} {{\textrm{d}}s \over {\textrm{d}}\phi }&={1 \over Lu^2}, \end{aligned}$$

2.18 $$\begin{aligned} {{\textrm{d}}\tau \over {\textrm{d}}\phi }&={CE \over Lu^{2}(1-2Mu)}, \end{aligned}$$

where

2.19 $$\begin{aligned} {\mathcal {F}}(u)\equiv & {} 2Mu^3-u^2-\left( \dfrac{2M}{L^2}\right) u + \left( \dfrac{1-C^{2}E^2}{L^2}\right) \nonumber \\= & {} 2M(u-u_1)(u-u_2)(u-u_3). \end{aligned}$$

The above differential equations completely determine the geodesic motion in Euclidean Schwarzschild geometry in the equatorial plane $\theta = \pi /2$. The turning points are described by the cubic equation

2.20 $$\begin{aligned} {\mathcal {F}}(u) =0, \end{aligned}$$

whose roots, say $u_1$, $u_2$ and $u_3$, satisfy the following equalities (Viète’s formulae):

2.21 $$\begin{aligned} u_1+u_2+u_3= & {} {1 \over 2M}, \end{aligned}$$

2.22 $$\begin{aligned} u_1 u_2 + u_2 u_3 + u_3 u_1= & {} -{1 \over L^2}, \end{aligned}$$

2.23 $$\begin{aligned} u_1 u_2 u_3= & {} -{\left( 1-C^{2}E^2\right) \over 2ML^2}. \end{aligned}$$

## Roots of the cubic equation ${\mathcal {F}}(u)=0$: qualitative analysis of the orbits

The cubic equation ${\mathcal {F}}(u)=0$ can be re-expressed in canonical form [34, 35]

3.1 $$\begin{aligned} w^{3}+pw+q=0, \end{aligned}$$

where

3.2 $$\begin{aligned} p&=-\left( {1 \over L^{2}}+{1 \over 12M^{2}}\right) , \end{aligned}$$

3.3 $$\begin{aligned} q&=-{1 \over 108M^{3}}+{1 \over 6ML^{2}}(2-3C^2E^{2}). \end{aligned}$$

Hence the discriminant $\bigtriangleup$ is given by

3.4 $$\begin{aligned} \bigtriangleup= & {} -(4p^{3}+27q^{2}) \nonumber \\= & {} {1 \over 4 (ML)^{2}} \left[ 16 \left( {M \over L}\right) ^{4} -(27C^4E^{4}-36C^2E^{2}+8)\right. \nonumber \\{} & {} \times \left. \left( {M \over L}\right) ^{2} +(1-C^2E^{2})\right] . \end{aligned}$$

From the above equations, it is clear that the integration constant *C* (cf. Eq. (2.7)) is a multiplicative constant and hence can be set to one without loss of generality. However, in order to keep our analysis as general as possible, we here continue employing a generic *C*.

**Fig. 1:**
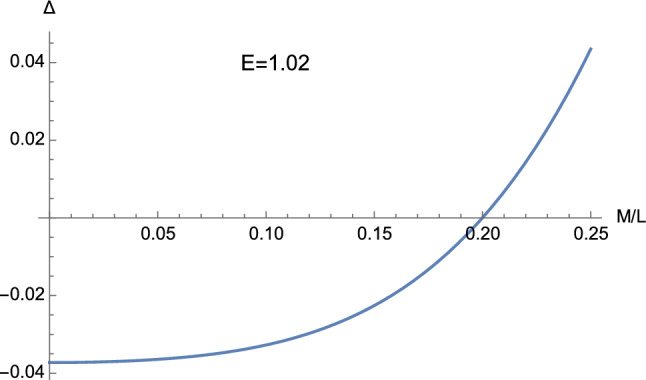
The discriminant (3.4) obtained with $E=1.02$ and $C=1$. It is clear that $\Delta$ assumes either positive, negative, or vanishing values

**Fig. 2:**
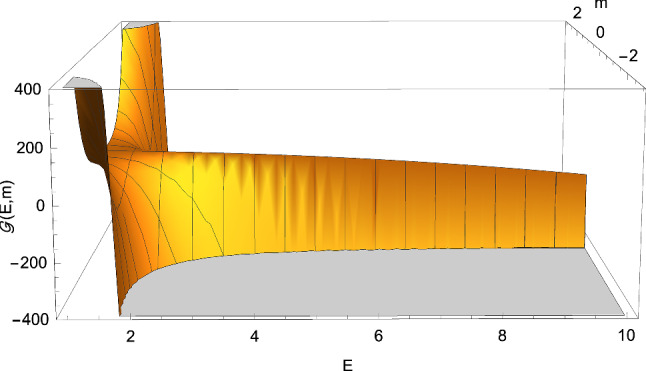
The function (3.5) with $C=1$. It is clear that the discriminant (3.4) can be either positive, negative, or zero if $C^2E^2 >1$ (cf. Eq. (3.7))

**Fig. 3:**
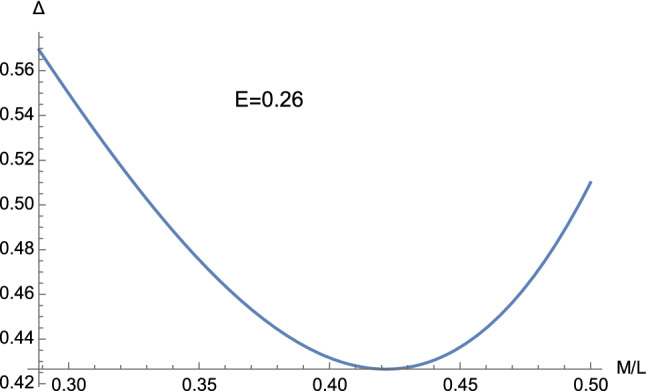
The discriminant (3.4) obtained with $E=0.26$ and $C=1$. It is clear that $\Delta$ never becomes negative

The sign of $\Delta$ depends on the behaviour of the real-valued function

3.5 $$\begin{aligned} {\mathscr {G}} \left( E,m\right)= & {} 16m^4 -\left( 27C^4E^4-36C^2E^2+8\right) m^2 \nonumber \\{} & {} +\left( 1-C^2E^2\right) , \end{aligned}$$

where

3.6 $$\begin{aligned} m \equiv \dfrac{M}{L}, \end{aligned}$$

the discriminant (3.4) being in fact expressible as

3.7 $$\begin{aligned} \Delta =\dfrac{1}{4\left( M^2 L\right) ^2} {\mathscr {G}} \left( E,m\right) . \end{aligned}$$

**Fig. 4:**
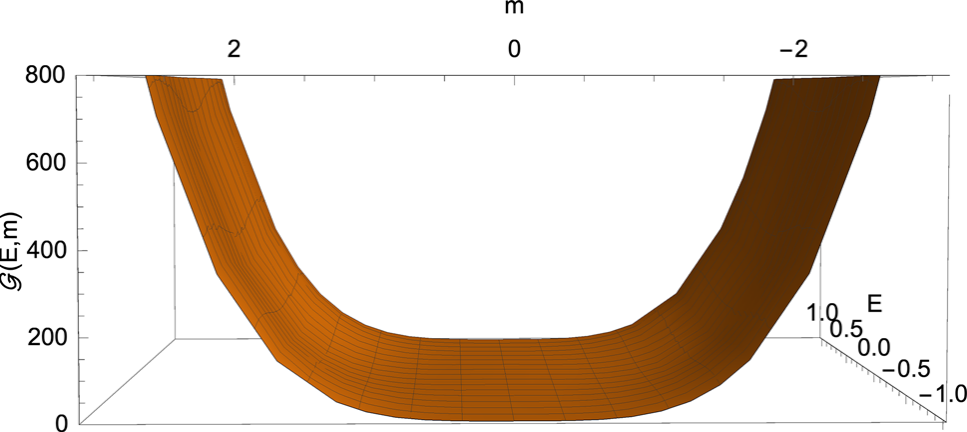
The function (3.5) with $C=1$. It is clear that the discriminant (3.4) never becomes negative provided $C^2E^2 \le 1$ (cf. Eq. (3.7))

In this way, we find that $\Delta$ can be either positive, negative, or zero only if $C^2E^2 >1$ (see Figs. 1 and 2), whereas when $C^2E^2 \le 1$ we only have $\Delta \ge 0$ (see Figs. 3 and 4). In particular, in this second case, $\Delta =0$ if

3.8a $$\begin{aligned} \vert m \vert&=\dfrac{1}{2}, \end{aligned}$$

3.8b $$\begin{aligned} E&=0, \end{aligned}$$

or

3.9a $$\begin{aligned} m&=0, \end{aligned}$$

3.9b $$\begin{aligned} CE&=1. \end{aligned}$$

This means that the cubic (2.20) can only admit real roots as soon as $C^2E^2 \le 1$. This is different from the Lorentzian case, where complex roots can arise both with $C^2E^2 >1$ and $C^2E^2 \le 1$.

**Fig. 5:**
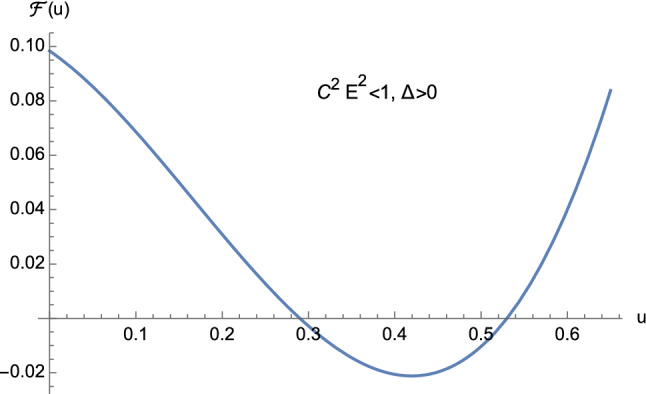
The positive roots of Eq. (2.20) when $C^2E^2<1$ and $\Delta >0$

**Fig. 6:**
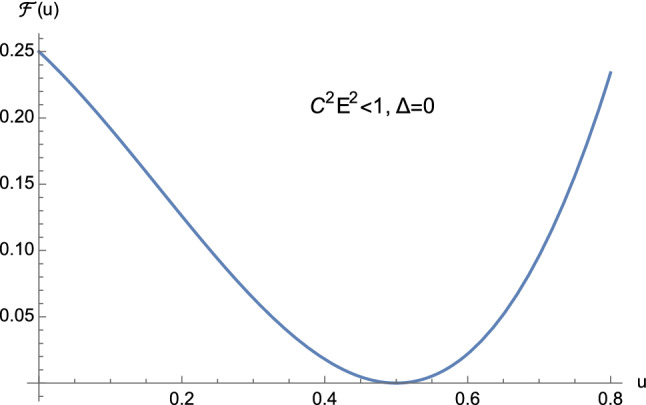
The positive roots of Eq. (2.20) when $C^2E^2<1$ and $\Delta =0$

**Fig. 7:**
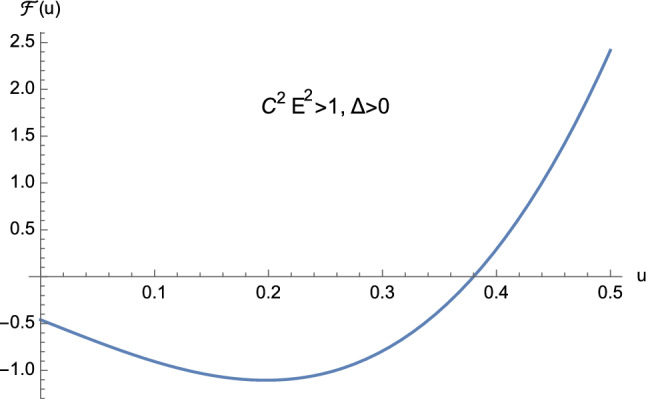
The positive root of Eq. (2.20) when $C^2E^2>1$ and $\Delta >0$

**Fig. 8:**
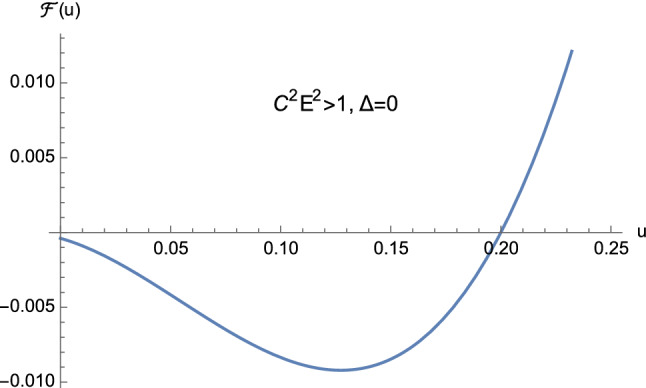
The positive root Eq. (2.20) when $C^2E^2>1$ and $\Delta =0$

**Fig. 9:**
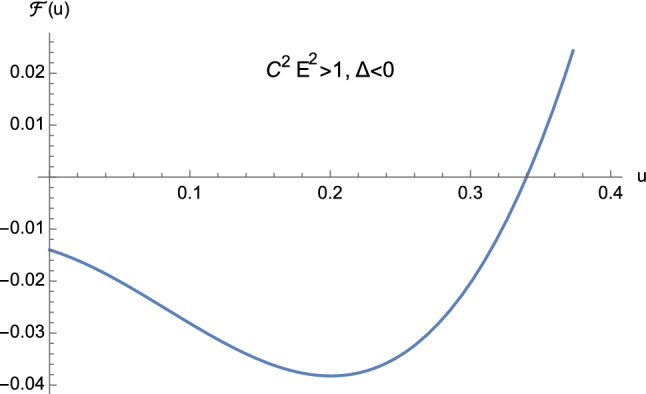
The positive root of Eq. (2.20) when $C^2E^2>1$ and $\Delta <0$

**Fig. 10:**
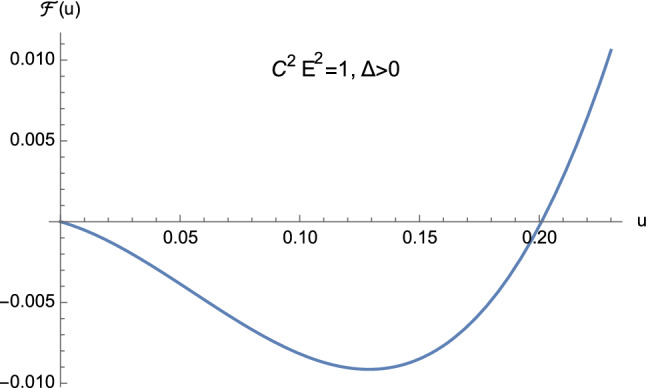
The non-negative roots of Eq. (2.20) when $C^2E^2=1$ and $\Delta >0$

**Fig. 11:**
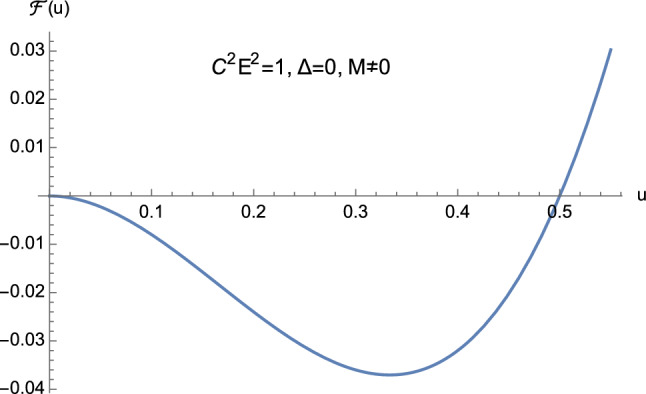
The roots of Eq. (2.20) when $C^2E^2=1$, $\Delta =0$, and $M \ne 0$

From the theory of cubic equations it is known that multiple roots arise when the discriminant (3.4) vanishes. In particular, if $\Delta =0$ and $p=0$, $w_1=w_2=w_3=0$ is a triple root of (3.1). On the other hand, if $\Delta =0$ and $p \ne 0$, then $w_1= 3q/p$ is a single root, while $w_2=w_3 = -3q/(2p)$ is a double root of (3.1). In our case, from Eq. (3.2) it is clear that $\Delta$ and *p* cannot vanish simultaneously. This means that the cubic equation (2.20) never admits a triple root when (3.4) vanishes. Furthermore, by employing Descartes’ rule of signs and bearing in mind the discriminant (3.4), we have the following situation:

- $$C^2E^2<1$$:
  (i) $\Delta >0$. The cubic (2.20) has two distinct positive roots and one negative root (see Fig. 5);
  (ii) $\Delta =0$. The cubic (2.20) admits one negative root and two coincident positive roots (see Fig. 6 and Eq. (3.8)) which read as
    3.10a $$\begin{aligned} u_1&=-\dfrac{1}{2M}, \end{aligned}$$
    3.10b $$\begin{aligned} u_2=u_3&=\dfrac{1}{2M}, \end{aligned}$$
    respectively.
- $$C^2E^2>1$$:
  (i) $\Delta >0$. The cubic (2.20) presents one positive root and two distinct negative roots (see Fig. 7);
  (ii) $\Delta =0$. The cubic (2.20) admits one positive root and two coincident negative roots (see Fig. 8);
  (iii) $\Delta <0$. The cubic (2.20) exhibits one positive root and two complex conjugate roots (see Fig. 9).
- $$C^2E^2 =1$$:
  (i) $\Delta >0$. The cubic (2.20) has one vanishing root, the negative root
    3.11 $$\begin{aligned} u_1=\dfrac{1-\sqrt{1+16\dfrac{M^2}{L^2}}}{4M} \end{aligned}$$
    and the positive root
    3.12 $$\begin{aligned} u_2=\dfrac{1+\sqrt{1+16\dfrac{M^2}{L^2}}}{4M}, \end{aligned}$$
    see Fig. 10;
  (ii) $\Delta =0$ with $M \ne 0$ and $\vert L\vert \gg M$ (see Eq. (3.9)). By virtue of Eqs. (3.11) and (3.12), we find that the cubic (2.20) admits a vanishing root (with multiplicity two) and the positive root $u_2=\dfrac{1}{2M}$ (see Fig. 11).

From Figs. 5, 6, 7, 8, 9, 10 and 11 it is clear that the conditions

$$\begin{aligned}&0<u_1<u<u_2, \nonumber \\&{\mathcal {F}}(u) > 0, \end{aligned}$$

never hold simultaneously. This is due to the fact that when (2.20) admits two positive roots (i.e., when $C^2E^2<1$) the function (2.19) is such that ${\mathcal {F}}(0) >0$. As a consequence, no elliptic-like orbits exist in Euclidean Schwarzschild geometry, unlike the corresponding Lorentzian pattern. Furthermore, since ${\mathcal {F}}(0) >0$ only if $C^2E^2<1$, we have the following classification:

3.13 $$\begin{aligned} C^2E^2&<1: \quad \text {unbounded orbits}, \end{aligned}$$

3.14 $$\begin{aligned} C^2E^2&>1: \quad \text {bounded orbits}, \end{aligned}$$

which amounts to the reversed situation with respect to general relativity. Here, bounded (resp. unbounded) orbits are defined as those trajectories where *r* remains bounded (resp. unbounded).

From Eq. (2.13a) we see that $\left( {\textrm{d}}r/{\textrm{d}}s\right) ^2 <0$ if $r=2M$. Therefore, the condition (2.2) should be tightened and for this purpose we impose

3.15 $$\begin{aligned} r>2M. \end{aligned}$$

In light of the above condition, the lower bound

3.16 $$\begin{aligned} r > \vert L \vert \end{aligned}$$

is a necessary but not sufficient condition to ensure that $\left( {\textrm{d}}r/{\textrm{d}}s \right) ^2 >0$.

Hereafter, we will limit our analysis to geodesics enforcing the constraint

3.17 $$\begin{aligned} u < \dfrac{1}{2M}, \end{aligned}$$

jointly with ${\mathcal {F}}(u) \ge 0$ (see Eq. (2.16)).

## Solution in terms of elliptic integrals

As we have shown before, the algebraic equation of third degree (2.20) involves three real roots as soon as $C^2E^2 \le 1$. In particular, when $C^2E^2<1$ and the discriminant (3.7) is non-vanishing, the solution $u_3$ turns out to admit the lower bound (see Appendix A for further details)

4.1 $$\begin{aligned} u_3 \ge \dfrac{1}{2M}, \end{aligned}$$

with $$u_3=1/(2M)$$ in the case $$E=0$$. On the other hand, Figs. 5 and 6 clearly indicate that the case $$C^2E^2<1$$ could, in principle, entail both first-kind trajectories, for which $$0< u\le u_2$$, and second-kind ones, where $$u>u_3 $$ (this is our definition of first-kind and second-kind orbits). Since the latter neither obey (3.15) nor belong to the real section of the complexified Schwarzschild spacetime, our calculations will be restricted to first-kind orbits. This represents a clear difference with respect to general relativity, where second-kind trajectories are always allowed.

Under the hypothesis $0<C^2E^2<1$, the three real solutions of the cubic (2.20) can be parametrised as

4.2a $$\begin{aligned} u_1&= -\dfrac{1}{\ell } \left( e-1\right) , \end{aligned}$$

4.2b $$\begin{aligned} u_2&= \dfrac{1}{2M} -\dfrac{2}{\ell }, \end{aligned}$$

4.2c $$\begin{aligned} u_3&= \dfrac{1}{\ell } \left( e+1\right) , \end{aligned}$$

where we have adopted a choice which does not resemble exactly the Lorentzian-signature framework [6] (see Appendix B for details).

The roots (4.2) clearly satisfy Eq. (2.21) and in addition

4.3 $$\begin{aligned} u_1<0<u_2<\dfrac{1}{2M}< u_3, \end{aligned}$$

provided that

4.4a $$\begin{aligned} \ell&>0, \end{aligned}$$

4.4b $$\begin{aligned} e&>1, \end{aligned}$$

4.4c $$\begin{aligned} \dfrac{1}{2\left( e+1\right) }<\mu&<\dfrac{1}{4}, \end{aligned}$$

where we have defined

4.5 $$\begin{aligned} \mu \equiv \dfrac{M}{\ell }. \end{aligned}$$

**Fig. 12:**
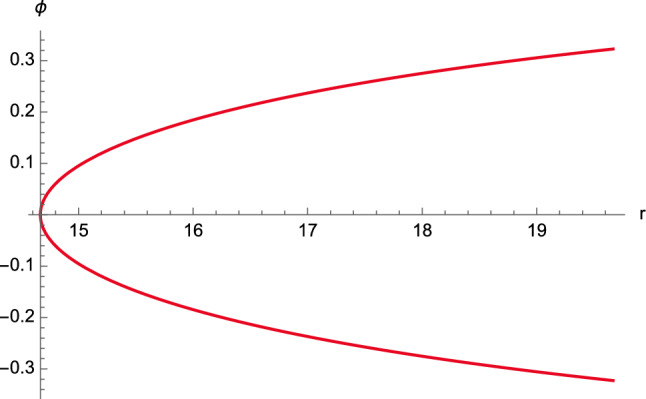
The function $\phi =\phi (r)$ for first-kind orbits having $C^2E^2<1$. The following constants have been chosen: $\phi _0=0$, $M=2$, $e=4.5$, $\ell =11$, $\varepsilon = \pm 1$, and $C=1$

**Fig. 13:**
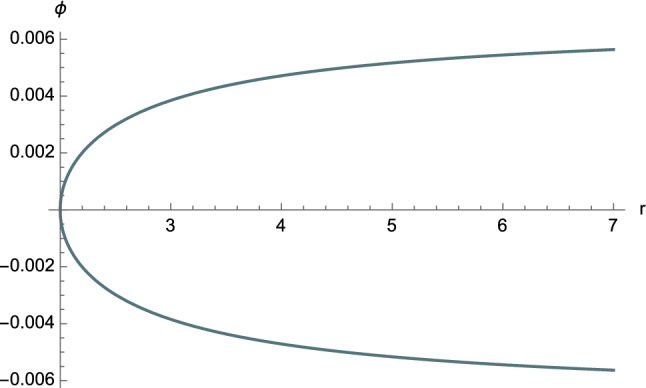
The function $\phi =\phi (r)$ for first-kind orbits having $C^2E^2<1$ in the limiting case (4.12). The following constants have been chosen: $\phi _0=0$, $M=1$, $e=1.5 \times 10^7$, $\ell =10^5$, $\varepsilon = \pm 1$, and $C=1$

It follows from Eqs. (4.4a) and (4.4b) that, similarly to the Lorentzian-signature pattern, we can interpret the positive constant $\ell$ as the *latus rectum* and *e* as the eccentricity; indeed, we will see that our investigation predicts the existence of trajectories which display a formal analogy with the hyperbolic orbits of general relativity (see Figs. 12 and 13, below).

Viète’s formulae (2.22) and (2.23) yield

4.6a $$\begin{aligned} \dfrac{1}{L^2}&= \dfrac{\mu \left( 3+e^2\right) -1}{M \ell }, \end{aligned}$$

4.6b $$\begin{aligned} \dfrac{\left( 1-C^2E^2\right) }{L^2}&= \dfrac{\left( e^2-1\right) \left( 1-4 \mu \right) }{\ell ^2}, \end{aligned}$$

respectively, and we recognize that the set of constraints (4.4) guarantees also that

4.7 $$\begin{aligned} L^2&>0, \end{aligned}$$

4.8 $$\begin{aligned} 0<C^2E^2&<1. \end{aligned}$$

In the hypothetical case

4.9 $$\begin{aligned} \mu = \dfrac{1}{\left( 6+2e\right) }, \end{aligned}$$

the roots (4.2b) and (4.2c) would coincide and relations (4.6) would be turned into

4.10a $$\begin{aligned} \dfrac{L^2}{M^2}&=\dfrac{4 \left( 3+e\right) ^2}{\left( e+1\right) \left( e-3\right) }, \end{aligned}$$

4.10b $$\begin{aligned} \left( 1-C^2E^2\right)&=\dfrac{\left( e^2-1\right) }{\left( e^2-9\right) }. \end{aligned}$$

However, Eqs. (4.2)–(4.4), as well as Eq. (4.8), do not account for this scenario. Indeed, we know that when $u_2=u_3$ both (3.8) and (3.10b) are satisfied, but the latter implies that the constraint (3.17) is violated, while, in light of the former, Eq. (4.10) cannot be valid; furthermore, it is clear that (3.8b) cannot stem from Eq. (4.8). Therefore, our analysis of first-kind trajectories naturally implies, on the one hand,

4.11 $$\begin{aligned} u_2 \ne u_3, \end{aligned}$$

while, on the other hand, it includes also the limiting situation

4.12 $$\begin{aligned} u_2 \rightarrow \dfrac{1}{2M} . \end{aligned}$$

First-kind orbits having $C^2E^2<1$ (i.e., unbounded, see Eq. (3.13)) will be dealt with in the following section.

### First-kind orbits having $C^2 E^2 <1$

As pointed out before, the case $C^2E^2<1$ consists of unbounded first-kind orbits only. This means that, equivalently, our study will rely on one portion of Fig. 5 only, whereas the situation depicted in Fig. 6 will be ignored.

Orbits of first kind are constrained by means of

4.13 $$\begin{aligned} 0<u \le u_2 <\dfrac{1}{2M}, \end{aligned}$$

see Fig. 5.

Starting from Eqs. (2.15)–(2.19), the system of differential equations for the geodesic motion can be re-expressed in the form (where $\varepsilon =\pm 1$)

4.14 $$\begin{aligned} {{\textrm{d}}\tau \over {\textrm{d}}s}&= {{\textrm{d}}\tau \over {\textrm{d}}r}{{\textrm{d}}r \over {\textrm{d}}s}={CE \over \left( 1-{2M \over r}\right) }, \end{aligned}$$

4.15 $$\begin{aligned} {{\textrm{d}}r \over {\textrm{d}}s}&=\varepsilon \,\sqrt{\left( 1-{2M \over r}\right) \left( 1-{L^{2}\over r^{2}}\right) -C^{2}E^{2}}, \end{aligned}$$

4.16 $$\begin{aligned} {{\textrm{d}}\phi \over {\textrm{d}}s}&={{\textrm{d}}\phi \over {\textrm{d}}r}{{\textrm{d}}r \over {\textrm{d}}s}={L \over r^{2}}, \end{aligned}$$

with the understanding that the physically relevant solution pertains to non-negative values of the argument of the square root on the right-hand side of Eq. (4.15). Moreover, as pointed out before, we focus on the case in which the root $u_{1}$ of the equation ${\mathcal {F}}(u)=0$ is negative, while the roots $u_{2}$ and $u_{3}$ are positive and such that (cf. Eqs. (4.3) and (4.13))

4.17 $$\begin{aligned} u \le u_{2} < u_{3}. \end{aligned}$$

We therefore find from Eqs. (4.14)–(4.16), upon setting $P_{3}(u)={\mathcal {F}}(u)/(2M)=(u-u_{1})(u-u_{2})(u-u_{3})$, the following integral formulae for the solution:

4.18 $$\begin{aligned} s&=s_{0}+{\varepsilon \over L \sqrt{2M}} \int _{{1 \over r}}^{u_{2}} {{\textrm{d}}u \over u^{2}\sqrt{P_{3}(u)}}, \end{aligned}$$

4.19 $$\begin{aligned} \tau&=\tau _{0}+{\varepsilon CE \over L \sqrt{2M}}\int _{{1 \over r}}^{u_{2}} {{\textrm{d}}u \over u^{2}(1-2Mu)\sqrt{P_{3}(u)}}, \end{aligned}$$

4.20 $$\begin{aligned} \phi&=\phi _{0}+{\varepsilon \over \sqrt{2M}} \int _{{1 \over r}}^{u_{2}} {{\textrm{d}}u \over \sqrt{P_{3}(u)}}. \end{aligned}$$

Note that, in agreement with what we said before, the upper limit of integration is $u_{2}$, in order to avoid negative values of $P_{3}(u)$, which are unphysical. At this stage, it is convenient to apply twice the method of adding and subtracting 2*Mu* in the numerator of the integrand in Eq. (4.19). Thus, upon defining (our $n=0,1,2$)

4.21a $$\begin{aligned} J_{n}&=\int _{{1 \over r}}^{u_{2}} {{\textrm{d}}u \over u^{n}\sqrt{P_{3}(u)}}, \end{aligned}$$

4.21b $$\begin{aligned} I&=\int _{{1 \over r}}^{u_{2}} {{\textrm{d}}u \over \left( u-{1 \over 2M}\right) \sqrt{P_{3}(u)}}, \end{aligned}$$

we obtain eventually the desired solution in the form

4.22 $$\begin{aligned} s&=s_{0}+{\varepsilon \over L \sqrt{2M}}J_{2}, \end{aligned}$$

4.23 $$\begin{aligned} \tau&=\tau _{0}+ CE(s-s_{0}) +\varepsilon CE{\sqrt{2M}\over L}(J_{1}-I), \end{aligned}$$

4.24 $$\begin{aligned} \phi&=\phi _{0}+{\varepsilon \over \sqrt{2M}}J_{0}. \end{aligned}$$

The four integrals occurring in the solution (4.22)–(4.24) can be evaluated by means of incomplete elliptic integrals (see Appendix C) according to the formulae [36]

4.25a $$\begin{aligned} a&=u_{3}, \quad b=u_{2}, \quad c=u_{1}, \end{aligned}$$

4.25b $$\begin{aligned} \varphi&=\text {arcsin} \sqrt{(a-c)\left( b-{1 \over r}\right) \over (b-c)\left( a-{1 \over r}\right) }, \end{aligned}$$

4.25c $$\begin{aligned} k^{2}&={(b-c)\over (a-c)}, \end{aligned}$$

4.25d $$\begin{aligned} \alpha ^{2}&={a \over b}k^{2}, \end{aligned}$$

4.25e $$\begin{aligned} \beta&=k \, \sqrt{{\left( {1 \over 2M}-a \right) \over \left( {1 \over 2M}-b \right) }}, \end{aligned}$$

4.26a $$\begin{aligned} J_{0}&={2 \over \sqrt{a-c}} F(\varphi ,k^{2}), \end{aligned}$$

4.26b $$\begin{aligned} J_{1}&={2 \over a \sqrt{a-c}} \left[ F(\varphi ,k^{2})+\left( {\alpha ^{2}\over k^{2}}-1 \right) \pi (\varphi ,\alpha ^{2},k^{2})\right] , \end{aligned}$$

4.26c $$\begin{aligned} J_{2}&= {2 \over a^{2}\sqrt{a-c}} \left\{ F(\varphi ,k^{2})+2\left( {\alpha ^{2}\over k^{2}}-1 \right) \pi (\varphi ,\alpha ^{2},k^{2}) \right. \nonumber \\&\quad + \left( {\alpha ^{2}\over k^{2}}-1 \right) ^{2} {1 \over 2 (\alpha ^{2}-1)(k^{2}-\alpha ^{2})} \Bigr [\alpha ^{2}E(\varphi ,k^{2}) \nonumber \\&\quad + (k^{2}-\alpha ^{2})F(\varphi ,k^{2}) +(2 \alpha ^{2}k^{2}+2\alpha ^{2}-\alpha ^{4}-3k^{2})\nonumber \\&\quad \times \pi (\varphi ,\alpha ^{2},k^{2}) - \left. {\alpha ^{4}\textrm{sn}(u)\textrm{cn}(u)\textrm{dn}(u) \over (1-\alpha ^{2}\textrm{sn}^{2}(u))} \Bigr ] \right\} , \end{aligned}$$

4.26d $$\begin{aligned} I&=-{2 \over (2M-a)\sqrt{a-c}}\nonumber \\&\quad \times \left[ F(\varphi ,k^{2})+\left( {\beta ^2 \over k^{2}}-1 \right) \pi (\varphi ,\beta ^2,k^{2})\right] . \end{aligned}$$

At a deeper level, the solution of Eq. (4.20) for ${1 \over r}=u(\phi )$ should not depend on the integration path. If one denotes by $\gamma$ a closed integration path and if one sets

4.27 $$\begin{aligned} {1 \over \sqrt{2M}} \int _{\gamma }{{\textrm{d}}u \over \sqrt{P_{3}(u)}}=\omega , \end{aligned}$$

this means that [7]

4.28 $$\begin{aligned} \phi -\phi _{0}-\omega =\frac{1}{\sqrt{2M}}\int _{u}^{u_{2}} {{\textrm{d}}u^\prime \over \sqrt{P_{3}(u^\prime )}}, \end{aligned}$$

should hold as well. In other words, the desired solution should be periodic of period $\omega$. At this stage, Eq. (4.20) is viewed as defined on the Riemann surface of the algebraic function $u \rightarrow \sqrt{P_{3}(u)}$. At the deep level of complex analysis and algebraic geometry, this is the appropriate concept of periodicity [7], which should not be confused with the periodicity of the function $y=\cos \left( \tfrac{\tau }{4M}\right) \sqrt{\tfrac{r}{2M}-1} \, \text {exp}\left( \tfrac{r}{4M}\right)$ in Kruskal–Szekeres coordinates [31].

### Graphical representation of unbounded first-kind orbits

Having obtained the general solution (4.22)–(4.24) of first-kind orbits that satisfy $C^2E^2<1$, we can now provide their graphical representation.

The plot of the solution $\phi =\phi (r)$ for unbounded first-kind orbits is displayed in Fig. 12, whereas the case of the limiting regime (4.12) is shown in Fig. 13. It is clear that the resulting trajectory has the same behaviour as the orbit displayed in Fig. 12.

It should be noted that the limiting scenario (4.12) is ruled by (cf. Eq. (4.2b))

4.29 $$\begin{aligned} \mu \rightarrow 0. \end{aligned}$$

By virtue of the constraint (4.4c), the condition (4.29) is admissible provided that (see Eq. (4.4b))

4.30 $$\begin{aligned} e \rightarrow + \infty , \end{aligned}$$

whereas the definition (4.5) of the parameter $\mu$ further demands (see Eq. (4.4a))

4.31 $$\begin{aligned} \ell \rightarrow + \infty . \end{aligned}$$

For the numerical evaluation of the inverse function $r=r(\phi )$, we refer the reader to the method in Sec. III of Ref. [7].

**Fig. 14:**
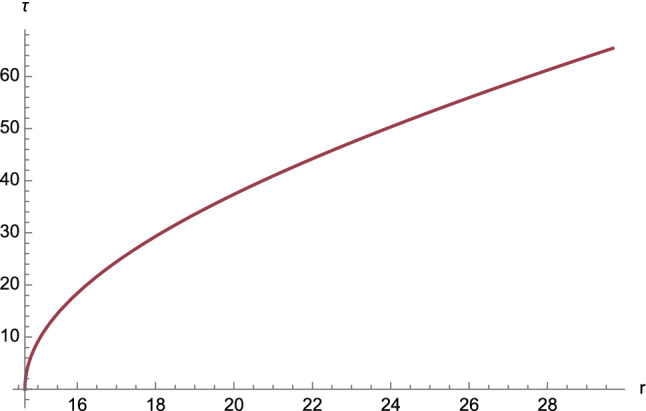
The function $\tau =\tau (r)$ for first-kind orbits having $C^2E^2<1$. The following constants have been chosen: $\tau _0=0$, $M=2$, $e=4.5$, $\ell =11$, $\varepsilon = \pm 1$, and $C=1$

**Fig. 15:**
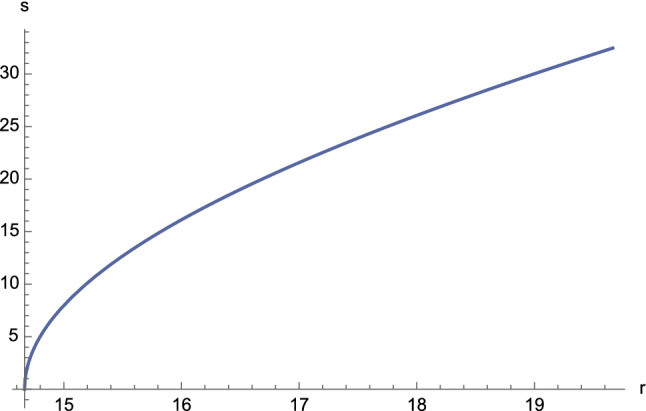
The function $s=s(r)$ for first-kind orbits having $C^2E^2<1$. The following constants have been chosen: $s_0=0$, $M=2$, $e=4.5$, $\ell =11$, $\varepsilon = \pm 1$, and $C=1$

The plots of the functions $\tau =\tau (r)$ and $s=s(r)$ are given in Figs. 14 and 15, respectively.

### Geodesics with $C^2E^2 \ge 1$

As pointed out before, as soon as $C^2E^2 >1$ the cubic (2.20) has only one positive root. We have checked that this solution is always bigger than 1/2*M* (see also Eq. (2.21)). Therefore, in view of the constraint (3.17), no geodesic motion is allowed when $C^2E^2 >1$. In other words, no bounded orbit exists in Euclidean Schwarzschild geometry.

The condition (3.17) demands that the case $C^2E^2=1$ entails only the root $u=0$. This means that when $C^2E^2=1$ the geodesic motion only allows $r=+\infty$.

## Lack of circular orbits

The last interesting topic to be addressed concerns the investigation of the possible presence of circular orbits. This task is performed in this section, where we will consider $C=1$ for simplicity.

By virtue of Eq. (2.13a), we can define an “Euclidean potential energy” $V_E(r)$ as

5.1 $$\begin{aligned} V_E(r)= \varepsilon \left( 1-\dfrac{2M}{r}\right) \left( 1-\dfrac{L^2}{r^2}\right) , \end{aligned}$$

where, as before, $\varepsilon =\pm 1$. It is known that [6] the minimum of the potential corresponds to a stable circular orbit, the maximum to an unstable one, whereas the point of inflection represents the innermost stable circular orbit. For the potential (5.1), we find that the first derivative

5.2 $$\begin{aligned} \dfrac{{\textrm{d}}V_E(r)}{{\textrm{d}}r}= \dfrac{2 \varepsilon }{r^4} \left[ M r^2 + L^2 (r-3M)\right] , \end{aligned}$$

vanishes at

5.3 $$\begin{aligned} r_{1,2} =\dfrac{-L^2 \mp \sqrt{L^4+12M^2 L^2}}{2M}. \end{aligned}$$

Since $r_1<0$, we will only consider the solution

5.4 $$\begin{aligned} r_2 \equiv r^\star . \end{aligned}$$

Then, from the study of the second derivative of $V_E(r)$, we obtain

5.5 $$\begin{aligned} \left. \dfrac{{\textrm{d}}^2 V_E(r)}{{\textrm{d}}r^2}\right| _{r^\star }=\dfrac{32 \varepsilon M^4 L^2 \left( L^2+12M^2 -\sqrt{L^4+12M^2L^2}\right) }{\left( \sqrt{L^4+12M^2L^2}-L^2\right) ^5}, \end{aligned}$$

which means that

**Fig. 16:**
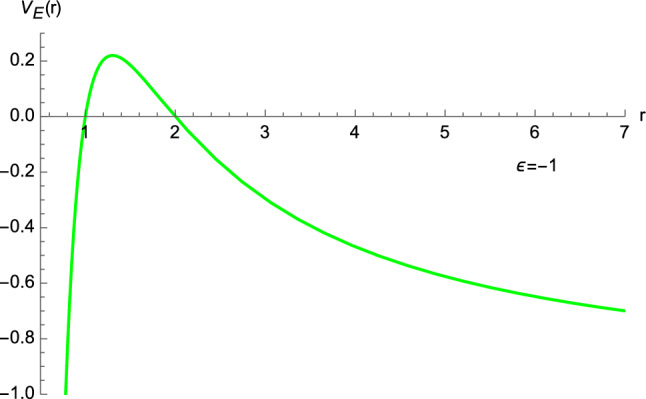
The potential energy function (5.1) with $\varepsilon =-1$, $M=1$, and $L=1$

**Fig. 17:**
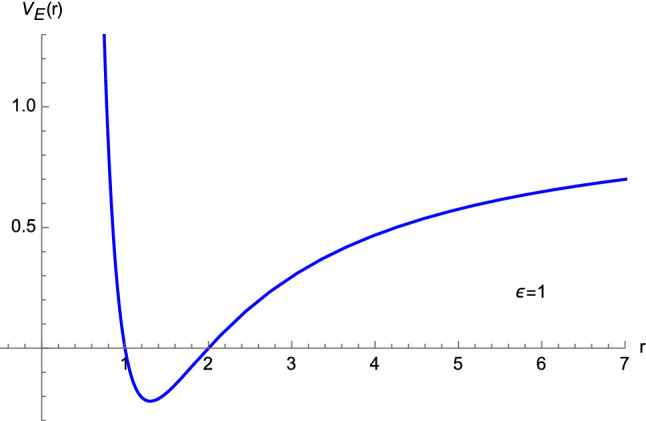
The potential energy function (5.1) with $\varepsilon =1$, $M=1$, and $L=1$

5.6 $$\begin{aligned}&r^\star \; \text{ is } \text{ a } \text{ maximum } \text{ of } V_E(r) \; \text{ if } \varepsilon =-1, \nonumber \\&r^\star \; \text{ is } \text{ a } \text{ minimum } \text{ of } V_E(r) \; \text{ if } \varepsilon =1, \end{aligned}$$

as shown in Figs. 16 and 17.

Furthermore, $r=r^\star$ cannot represent a point of inflection since the condition $\left. \tfrac{{\textrm{d}}^2 V_E(r)}{{\textrm{d}}r^2} \right| _{r^\star }=0$ implies $12M^2 + L^2=0$, which in turn does not lead to any real-valued solution. Despite the result (5.6), no circular orbit can exist in our model (even if $r^\star >2M$ when $\vert L \vert >2M$). In fact, bearing in mind Eq. (2.13a), we see that the requirement $\left( \tfrac{{\textrm{d}}r}{{\textrm{d}}s}\right) ^2 >0$ entails, when $E^2>1$,

5.7 $$\begin{aligned} \dfrac{V_E(r)}{\varepsilon } >1, \end{aligned}$$

but this lower bound is not fulfilled at $r=r^\star$. Furthermore, as a consequence of Eq. (2.13a), the condition $\left( \tfrac{{\textrm{d}}r}{{\textrm{d}}s}\right) ^2 =0$ yields

5.8 $$\begin{aligned} \dfrac{V_E(r)}{\varepsilon } =E^2, \end{aligned}$$

which, when evaluated at $r=r^\star$, leads to complex-valued solutions for the energy *E* (equivalently, these solutions do not satisfy $E^2>1$ nor do they fulfill $0<E^2<1$).^1^ Since circular orbits are not present also if $E^2\le 1$, this completes our proof that Euclidean Schwarzschild geometry does not envisage circular orbits. This differs from general relativity, where both stable and unstable circular trajectories are predicted, the innermost stable circular orbit occurring at $r=6M$ [6].

We have been looking for circular geodesics that make a loop around the Euclidean time and correspond to constant values of *r* and $\phi$. However, when *r* and $\phi$ are constant, Eq. (2.3c) is solved for $\tau (\uplambda )$ by a linear function of the affine parameter, while Eq. (2.3a) shows that ${\textrm{d}} \tau / {\textrm{d}} \uplambda =0$. Thus, the Euclidean time $\tau$ is found to be constant, and the desired circular geodesic shrinks to the point

5.9 $$\begin{aligned} (\tau = \text {constant}, r=\text {constant}, \phi =\text {constant}). \end{aligned}$$

## Conclusions

In this paper we have evaluated in detail geodesic motion in Euclidean Schwarzschild geometry, limited to the real Riemannian section of the complexified Schwarzschild spacetime. Our explicit solution (4.22)–(4.24) in terms of incomplete elliptic integrals of first, second and third kind has never appeared in the literature, to the best of our knowledge.

Our investigation has revealed new interesting features, which do not occur in the corresponding Lorentzian-signature framework. This means that the Euclidean and the Lorentzian Schwarzschild geometries are characterized by deep differences which cannot be merely reduced to the opposite signs occurring in the timelike component of their metric tensors. Indeed, we have shown that no elliptic-like orbits occur in the Euclidean Schwarzschild spacetime and, in general, bounded orbits are not allowed. Furthermore, unbounded orbits consist of first-kind trajectories only and are described by means of a parametrization which differs from the one adopted in general relativity (see Eq. (4.2)).

Recently, a new examination of the geodesic motion in Lorentzian Schwarzschild geometry has been proposed in the literature, where all kinds of nonradial causal geodesic orbits have been described via a single formula making use of Weierstrass elliptic functions [37]. On the other hand, the Euclidean case studied in this paper exploits incomplete elliptic integrals. Thus, an interesting issue to be addressed could consist in verifying whether the pattern of Ref. [37] can be employed also in Euclidean settings.

The lack of bounded orbits in Euclidean Schwarzschild geometry is a feature existing also at quantum level. Indeed, it has been shown in Ref. [30] that only the inclusion of a “magnetic field” (i.e., a self-dual Abelian gauge field) yields bounded (elliptic) orbits (the same conclusions hold also for Taub-NUT and Taub-Bolt spaces, see Refs. [38, 39]). Moreover, in this framework (and in particular in the context of the recently proposed geometric models of matter [28]) the Euclidean Schwarzschild space emerges as a natural geometric candidate for the neutron [29] (whereas the Euclidean Taub-NUT space can represent the electron [28]).

The investigation of singularities in Euclidean Schwarzschild geometry is a physical motivation supporting our paper. In fact, it is known [40] that in general relativity timelike and null geodesic incompleteness is the criterion used to define the occurrence of space-time singularities. On the other hand, in the case of Euclidean Schwarzschild geometry, the absence of the singularity at $r=0$ is demonstrated via a “shortcut” by considering the real section of the complexified Schwarzschild spacetime in Kruskal–Szekeres coordinates [31]. Our analysis can be thus exploited to show that the geodesics of (the real section of) the Euclidean Schwarzschild spacetime are indeed complete and hence no singularity can emerge.

Last, this work can represent a starting point for a systematic study of geodesic motion in Euclidean gravity. Thus, the first step carried out in this paper can be followed by an analysis involving the whole set of gravitational instantons in general. This might entail the discovery of new results both in Riemannian geometry and Euclidean quantum gravity.

## Data Availability

This manuscript has no associated data or the data will not be deposited. [Authors’ comment: Data sharing not applicable to this article as no new data were created or analyzed in this study.]

## Appendix A: General formulae for roots of the cubic ${\mathcal {F}}(u)=0$

The three roots of the cubic (2.20) can be obtained by means of a *numerical* evaluation of the following quantities (recall that $m \equiv M/L$):

A1 $$\begin{aligned} u_1&=\dfrac{1}{6M} +\dfrac{\left( 1+ \textrm{i} \sqrt{3}\right) \left( 1+12m^2\right) }{\left( 6M \root ^{3} \of {4} \right) {\mathscr {B}}} +\dfrac{\left( -1+\textrm{i}\sqrt{3}\right) {\mathscr {B}}}{12M \root ^{3} \of {2}}, \end{aligned}$$

A2 $$\begin{aligned} u_2&=\dfrac{1}{6M} -\dfrac{\left( 1- \textrm{i} \sqrt{3}\right) \left( 1+12m^2\right) }{\left( 6M \root ^{3} \of {4} \right) {\mathscr {B}}} -\dfrac{\left( 1+\textrm{i}\sqrt{3}\right) {\mathscr {B}}}{12M \root ^{3} \of {2}}, \end{aligned}$$

A3 $$\begin{aligned} u_3&= \dfrac{1}{6M} +\dfrac{\left( 1+12m^2\right) }{\left( 3M \root ^{3} \of {4} \right) {\mathscr {B}}} +\dfrac{{\mathscr {B}}}{6M \root ^{3} \of {2}}, \end{aligned}$$

where

A4 $$\begin{aligned} {\mathscr {B}} \equiv \root ^{3}3 \of {\left( 2-72m^2+108C^2E^2 m^2 \right) +\sqrt{\left( 2-72m^2+108C^2E^2m^2\right) ^2-4 \left( 1+12m^2\right) ^3}}. \end{aligned}$$

The above formulae are valid for any real-valued *C* and *E*.

## Appendix B: More details about the roots of the cubic ${\mathcal {F}}(u)=0$ under the hypothesis $C^2E^2<1$

In Sect. 4, we have seen that the form (4.2) of the roots of the cubic (2.20) accounts correctly for the geodesic motion under the hypothesis $C^2E^2<1$. In this Appendix we will show that, had we chosen the same parametrization as in general relativity [6], we would have obtained some inconsistencies. Let

B1a $$\begin{aligned} u^\prime _1&= -\dfrac{1}{\ell } \left( e-1\right) , \end{aligned}$$

B1b $$\begin{aligned} u^\prime _2&= \dfrac{1}{\ell } \left( e+1\right) , \end{aligned}$$

B1c $$\begin{aligned} u^\prime _3&= \dfrac{1}{2M} -\dfrac{2}{\ell }, \end{aligned}$$

denote a new (hypothetical) set of roots of the cubic (2.20) in the case $C^2E^2<1$. Equation (B1) is identical to the choice adopted in the context of Einstein’s theory [6] and furthermore it is clear that (cf. Eq. (4.2)) $u^\prime _1=u_1$, $u^\prime _2=u_3$, and $u^\prime _3=u_2$. In addition, by means of the parametrization (B1), the constraint (4.3) is not enforced since

B2 $$\begin{aligned} u^\prime _1<0<u^\prime _2< u^\prime _3 <\dfrac{1}{2M}, \end{aligned}$$

provided that

B3a $$\begin{aligned} \ell&>0, \end{aligned}$$

B3b $$\begin{aligned} e&>1, \end{aligned}$$

B3c $$\begin{aligned} \mu&<\dfrac{1}{4}, \end{aligned}$$

B3d $$\begin{aligned} 1-6\mu -2\mu e&> 0, \end{aligned}$$

where $\mu$ has been defined in Eq. (4.5). Condition (B3c) is guaranteed by (B3b) and (B3d). Viète’s formulae (2.22) and (2.23) lead to the same relations as in Eq. (4.6), i.e.,

B4a $$\begin{aligned} \dfrac{1}{L^2}&= \dfrac{\mu \left( 3+e^2\right) -1}{M \ell }, \end{aligned}$$

B4b $$\begin{aligned} \dfrac{\left( 1-C^2E^2\right) }{L^2}&= \dfrac{\left( e^2-1\right) \left( 1-4 \mu \right) }{\ell ^2}. \end{aligned}$$

Equations (B3d) and (B4a) entail

B5 $$\begin{aligned} e>3, \qquad \dfrac{1}{\left( 3+e^2\right) }< \mu < \dfrac{1}{\left( 6+2e\right) }, \end{aligned}$$

and hence the set of constraints (B3) should be slightly modified according to

B6a $$\begin{aligned} \ell&>0, \end{aligned}$$

B6b $$\begin{aligned} e&>3, \end{aligned}$$

B6c $$\begin{aligned} \mu&<\dfrac{1}{4}, \end{aligned}$$

B6d $$\begin{aligned} \dfrac{1}{\left( 3+e^2\right) }&< \mu < \dfrac{1}{\left( 6+2e\right) }, \end{aligned}$$

where (B6c) is fulfilled on account of (B6b) and (B6d). Note that, unlike the analysis of Sect. 4, the parameter *e* is such that $e \notin (1,3)$ (cf. Eqs. (4.4b) and (B6b)). At this stage, from the requirement

B7 $$\begin{aligned} E^2>0, \end{aligned}$$

we obtain from Eq. (B4)

B8 $$\begin{aligned} \mu > \dfrac{1}{2\left( e+1\right) }, \end{aligned}$$

which disagrees with Eq. (B6d). Thus, as we have seen in Sect. 4, the parametrization (4.2) should be employed in place of (B1).

## Appendix C: Incomplete elliptic integrals

According to the standard notation in Ref. [36], the incomplete elliptic integrals of the first, second, and third kind can be defined as

C1 $$\begin{aligned} F(\varphi ,k^{2})&=\int _{0}^{\varphi } {{\textrm{d}}\theta \over \sqrt{1-k^{2}\sin ^{2}\theta }}, \end{aligned}$$

C2 $$\begin{aligned} E(\varphi ,k^{2})&=\int _{0}^{\varphi } \sqrt{1-k^{2}\sin ^{2}\theta } \; {\textrm{d}}\theta , \end{aligned}$$

C3 $$\begin{aligned} \pi (\varphi ,\alpha ^{2},k^{2})&=\int _{0}^{\varphi } {{\textrm{d}}\theta \over (1-\alpha ^{2}\sin ^{2}\theta ) \sqrt{1-k^{2}\sin ^{2}\theta }}, \end{aligned}$$

respectively, while the Jacobi elliptic functions read as

C4 $$\begin{aligned} \textrm{sn}(u)=\sin \varphi , \quad \textrm{cn}(u)=\cos \varphi , \quad \textrm{dn}(u)=\sqrt{1-k^{2}\sin ^{2}\varphi }. \nonumber \\ \end{aligned}$$

## Footnotes

1 The equation $\tfrac{V_E(r^\star )}{\varepsilon } =E^2$ leads also to the real-valued solution $E=0$ when $\vert L \vert =2M$. However, this solution cannot be accepted for two reasons: (i) it violates Eq. (2.14a); (ii) it does not fulfill the constraint $r^\star >2M$.
